# Frailty and Energy Intake Deficiency Reduce the Efficiency of Activities of Daily Living in Patients with Musculoskeletal Disorders: A Retrospective Cohort Study

**DOI:** 10.3390/nu17081334

**Published:** 2025-04-12

**Authors:** Yusuke Tamamura, Chihiro Hachiuma, Michiko Matsuura, Sumiko Shiba, Toshio Nishikimi

**Affiliations:** 1 Department of Rehabilitation, Wakakusa-Tatsuma Rehabilitation Hospital, 1580 Ooaza Tatsuma, Daito 574-0012, Osaka, Japan; yt.tatsuma3586@gmail.com (Y.T.); wakatatsu.mmp@gmail.com (M.M.); 2 Department of Nutrition, Wakakusa-Tatsuma Rehabilitation Hospital, 1580 Ooaza Tatsuma, Daito 574-0012, Osaka, Japan; akkgd928@gmail.com; 3 Department of Physical Therapy, Konan Women’s University, 6-2-23 Morikita-cho, Higashinada-ku, Kobe 658-0001, Hyogo, Japan; s.shiba@konan-wu.ac.jp; 4 Department of Medicine, Wakakusa-Tatsuma Rehabilitation Hospital, 1580 Ooaza Tatsuma, Daito 574-0012, Osaka, Japan

**Keywords:** clinical frailty scale, energy intake, musculoskeletal disorders, rehabilitation, activities of daily living, convalescent rehabilitation wards

## Abstract

**Background/Objective:** This study aimed to investigate the relationship between rehabilitation effectiveness (RE) and pre-admission Clinical Frailty Scale (CFS) scores and energy intake. **Methods:** This retrospective observational study included 735 patients (81 ± 10 years; male: 27.5%) with musculoskeletal disorders discharged from convalescent rehabilitation wards between April 2018 and April 2024. The patients were classified into four groups based on their CFS scores (non-frail, CFS 1–3; frail, CFS ≥ 4) and rate of energy intake (energy-sufficient vs. energy-deficient). Group comparisons of RE were conducted, and the relationships between the CFS score, energy intake, and RE were analyzed. **Results:** The RE was significantly lower in the frail/energy-deficient group (53.6 [41.9–78.1]) than in the non-frail/energy-sufficient (78.5 [61.8–90.7]), non-frail/energy-deficient (70.6 [53.4–87.4]), and frail/energy-sufficient (59.9 [41.9–78.1]) groups. Additionally, the frail/energy-sufficient group had significantly lower RE scores than the non-frail/energy-sufficient and non-frail/energy-deficient groups. A multiple linear regression analysis revealed that age, male sex, CFS score, energy intake, handgrip strength, Functional Oral Intake Scale score, Mini Nutritional Assessment-Short Form score, B-type natriuretic peptide, and creatinine were significantly associated with the RE. **Conclusions:** Both frailty and inadequate energy intake reduce the rate of improvement in activities of daily living in patients with musculoskeletal diseases.

## 1. Introduction

With the aging of populations in many countries, health issues specific to the elderly have become increasingly important. Among these, musculoskeletal disorders are major concerns that significantly affect the quality of life and functional independence of older adults [1]. According to a national survey in Japan, the estimated prevalence of musculoskeletal disorders in the elderly (age > 65 years) is 50–80% for knee osteoarthritis and 75–90% for lumbar osteoarthritis [2]. The functional decline caused by bone fractures and musculoskeletal diseases in the elderly significantly reduces their ability to perform activities of daily living (ADLs) [3].

In recent years, frailty has also been attracting attention as a factor that reduces the ability to perform ADLs [4]. Frailty is a biological syndrome characterized by a high vulnerability to low-power stressors, manifested clinically by decreased functional reserve and resilience, together with multiorgan dysfunction or multimorbidity [5]. The prevalence of frailty among individuals admitted to geriatric rehabilitation is as high as 66% [6,7], and frailty is associated with poor health outcomes characterized by functional decline and institutionalization [8,9]. Previous studies have demonstrated that frailty, as measured by tools such as the Clinical Frailty Scale (CFS), is associated with an increased length of hospital stays, difficulty in being discharged home, and increased late mortality [10,11,12].

In addition to frailty, energy intake has emerged as a critical factor influencing rehabilitation outcomes. For stroke patients with a mean age in the 60s, inadequate energy intake was common even among patients who were less impaired and relatively independent at admission, and the dietary energy intake was predictive of rehabilitation outcomes in these patients [13]. Therefore, the early assessment of malnutrition risk in post-stroke patients may help prevent further functional decline and contribute to optimizing rehabilitation outcomes. Moreover, the amount of energy intake at admission was positively associated with the recovery of ADLs in malnourished stroke patients with a mean age in the 60s [14]. Similarly, the nutritional status is also related to ADL recovery in patients with musculoskeletal diseases with a mean age in the 80s [15,16,17]. Thus, nutritional intake is essential for improving ADLs through rehabilitation; however, few studies have calculated an individual’s required energy intake or examined actual rates of energy intake. If an individual’s voluntary energy intake does not meet their estimated energy requirements, it may result in accelerating malnutrition and delayed functional recovery even with full intake. These results indicate that in addition to the individual’s calculated total energy intake requirements, the actual energy intake rate for providing energy is also important.

Although the effect of frailty and nutritional intake are recognized as important factors affecting rehabilitation outcomes [18], few studies have explored the combined effects of these factors, particularly in patients with musculoskeletal disorders. Therefore, this study aims to investigate the effect of frailty, as assessed by the CFS, and the actual rate of energy intake relative to the calculated total required energy on the efficiency of ADL improvement in patients with musculoskeletal disorders in convalescent rehabilitation wards.

## 2. Materials and Methods

### 2.1. Ethics

This study adhered to the Declaration of Helsinki and was approved by the Wakakusa-Tatsuma Rehabilitation Hospital Ethics Committee (approval number: 19100761). The requirement for informed consent was waived because this study was a retrospective analysis of routinely collected data. Furthermore, information about the study was disclosed on the bulletin board, and an opt-out procedure enabled patients to deny use of their medical information.

### 2.2. Participants and Setting

**Figure 1:**
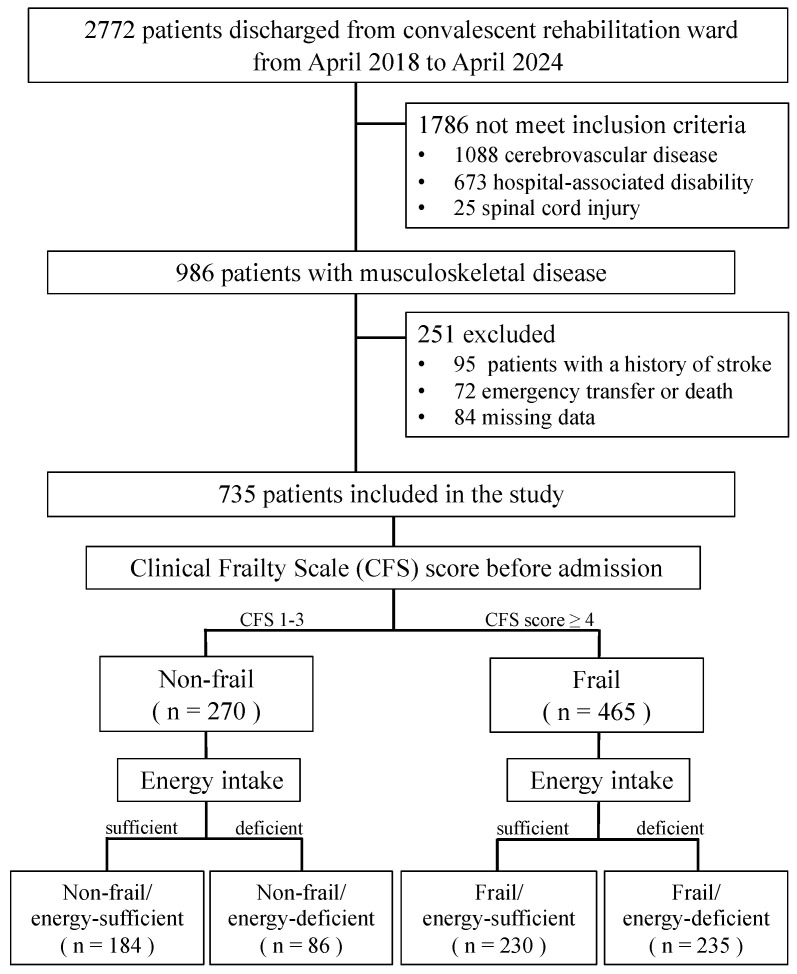
Flow chart of this study.

This retrospective cohort study was conducted in convalescent rehabilitation wards at the Wakakusa-Tatsuma Rehabilitation Hospital in Japan. Initially enrolled were 2772 consecutive patients admitted to convalescent rehabilitation wards between April 2018 and April 2024 and subsequently discharged. Among these patients, patients with stroke (*n* = 1088), hospitalization-associated disability (*n* = 673), or spinal cord injury (*n* = 25) were excluded. Patients with musculoskeletal disorders (*n* = 986) were selected for further analysis. From that group, we excluded patients with a history of stroke (*n* = 95), those who required emergency transfer or died during hospitalization (*n* = 72), and those with missing data (*n* = 84). Ultimately, 735 patients were included in the study (Figure 1).

In convalescent rehabilitation wards in Japan, public medical insurance covers patients’ individual rehabilitation provided by physical therapists, occupational therapists, and speech–language–hearing therapists for a maximum of nine units per day (1 unit = 20 min), 7 days per week. The time allocation for each type of rehabilitation is tailored to the needs of the individual patient. In this study, patients underwent a rehabilitation program that included conventional physical therapy and occupational therapy performed for 6 to 8 units per day according to the patient’s condition until discharge. Physical therapists performed active-assisted and active mobilizations; exercises for muscle strength recovery, body position change, and transfers; sitting-and-standing training; motor coordination and balance training; and walking training. Occupational therapists assessed the patient’s home environment and performed ADL training appropriate for the living environment.

Basic information, including age, sex, height, weight, body mass index (BMI), comorbidities (such as hypertension, diabetes mellitus, dyslipidemia, and atrial fibrillation), and medications at admission were collected from medical records. Clinical data, including results of physical examinations, swallowing function tests, blood tests, and ADL measurements, were also collected. The physical examinations included handgrip strength and quadriceps strength.

Swallowing function was evaluated using the Functional Oral Intake Scale (FOIS) [19]. Nutritional status was assessed based on the Mini Nutritional Assessment-Short Form (MNA-SF) [20] and Geriatric Nutritional Risk Index (GNRI) [21]. These nutritional indicators are useful screening tools that have been validated in several studies of patients admitted to convalescent rehabilitation wards [22,23]. In addition, thigh and calf circumferences were measured to further assess muscle mass and nutritional status. Blood tests were performed to evaluate serum albumin, *C*-reactive protein (CRP), creatinine, total cholesterol, and hemoglobin. B-type natriuretic peptide (BNP) was also measured using a commercial immunoassay.

ADL was evaluated using the Barthel Index (BI) [24] and FIM instrument [25].

### 2.3. Assessment of Frailty and Energy Intake

The degree of frailty was determined using the Japanese version of the CFS, translated by The Japan Geriatrics Society in 2021 (Supplemental Figure S1). The CFS is a comprehensive index for evaluating the degree of frailty on a 9-point scale, as proposed by Rockwood et al. [26]. The scale allocates a high score for decline in both physical and cognitive functions, taking into account an individual’s level of independence in ADLs and their need for nursing care. The evaluation does not require specialized equipment or take a long period of time, which makes the CFS is a useful index that enables comprehensive evaluation based on clinical findings [27]. Based on previous studies using the CFS, which defined CHF 1–3 as non-frail [12,28,29,30], we divided the patients into two groups according to their pre-hospital CFS: non-frail (CFS 1–3) and frail (CFS 4–9).

Energy intake was assessed by dietitians. Individual caloric requirements were calculated using the Harris–Benedict equation (HBE) [31] multiplied by an activity factor. The HBE is considered to be the best tool for predicting basal energy expenditure (BEE) [32]. The calculated BEE must then be adjusted using an activity factor to predict the patient’s total energy requirements for life. The activity factors ranged from 1.2 (sedentary) to 1.5 (moderate activity) and were determined by the attending physician, dietitian, and physical therapist based on the patient’s condition and the planned rehabilitation program. For patients with sufficient energy intake, the activity factor was set at 1.5, and they underwent gait training and ADL practice. In contrast, for patients with insufficient energy intake, the activity factor was set at 1.2, and bedside range of motion (ROM) exercises and muscle strengthening exercises were performed (Supplemental Table S1).

Oral energy intake was measured by the nurses using the visual estimation method, and further calculations were carried out by registered dietitians. The visual estimation method is commonly used in hospitals and other care facilities to evaluate food intake through the estimation of plate waste [33]. The visual estimation method was carried out by the nurses and consisted of first visually estimating the food present before the tray was provided to the patient. After the patient finished the meal, a nurse would register the intake of grain and other foods on diet records using an 11-point scale of 0–10. The enteral and parenteral nutrition and the total energy intake were recorded by registered nutritionists. In this study, nutritional intake was defined as the average energy intake over 3 days after hospitalization, and the ratio of actual intake to the provided energy intake was calculated.

Patients were categorized into two groups based on their daily ratio of energy intake. Those whose energy intake per day reached the amount of energy provided were designated the energy-sufficient group, while those whose energy intake per day did not reach the amount of energy provided were designated as the energy-deficient group. The patients were then further divided into four groups according to their CFS and energy intake: (1) non-frail/energy-sufficient group, (2) non-frail/energy-deficient group, (3) frail/energy-sufficient group, and (4) frail/energy-deficient group (Figure 1).

### 2.4. Rehabilitation Outcome

The primary rehabilitation outcome was rehabilitation effectiveness (RE). RE was calculated using the FIM instrument and the following formula: (FIM at discharge/FIM at admission)/(126 − FIM at admission) × 100%. By expressing RE as a percentage reflecting the proportion of potential improvement actually achieved during rehabilitation, RE was corrected for a ceiling effect [34]. Earlier studies have reported the use of RE as a primary outcome in rehabilitation research, and its validity has been well established [22,23,35]. The secondary outcome was FIM efficiency during hospitalization in the ward. FIM efficiency was calculated as follows: (FIM score at discharge − FIM score on admission)/length of hospital stay.

### 2.5. Sample Size Calculation

The sample size for this study was determined based on a power analysis for a one-way analysis of variance (ANOVA). Assuming a standard deviation of 38 for RE and a mean difference of 15 between the energy-sufficient group and the energy-deficient group, as reported in a previous study [36], with a power of 0.80 and a significance level (α) of 0.05, the analysis indicated that a minimum of 79 participants per group was required. Given the study design with four groups, the total required sample size was calculated to be at least 316 participants. To ensure the robustness of the results, we recruited 735 participants, resulting in group sizes of 184, 86, 230, and 235 participants, respectively.

### 2.6. Statistical Analysis

Continuous data are presented as the mean ± standard deviation and non-parametric data as the median (interquartile range 25–75 percentile). Differences among groups were evaluated using one-way ANOVA followed by Bonferroni post hoc tests. Differences in RE among groups were assessed using analysis of covariance (ANCOVA) adjusted for age and gender. Categorical data are expressed as incidences and percentages, and comparisons were made using the chi-square test. The interaction effect between frailty, assessed using the CFS and energy intake ratio, on RE was also examined. In addition, we performed univariate and multiple regression analyses with RE as the dependent variable and factors that showed a significant correlation with RE. Multicollinearity among factors was assessed using the variance inflation factor (VIF) and was considered present when the VIF was ≥2. Values of *p* < 0.05 were considered statistically significant. Statistical analyses were performed using SPSS version 29.0 (IBM, Armonk, NY, USA).

## 3. Results

Among the 735 subjects, 184 were assigned to the non-frail/energy-sufficient group, 86 to the non-frail/energy-deficient group, 230 to the frail/energy-sufficient group, and 235 to the frail/energy-deficient group. The demographic and baseline clinical characteristics of each group are shown in Table 1. The frail/energy-deficient group was significantly older than the other three groups and had a lower BMI than the non-frail/energy-sufficient group. Regarding complications, the prevalence of diabetes mellitus was significantly lower in the frail/energy-deficient group than the non-frail/energy-sufficient group; however, there were no significant differences in the prevalence of hypertension, dyslipidemia, or atrial fibrillation.

The handgrip strength was significantly lower in the frail/energy-deficient group than the other three groups. It was also significantly lower in the non-frail/energy-deficient group and the frail/energy-sufficient group than in the non-frail/energy-sufficient group. The quadriceps strength was significantly lower in the frail/energy-deficient group than in the non-frail/energy-sufficient group. The FOIS score was significantly lower in the frail/energy-deficient group than in the non-frail/energy-sufficient and non-frail/energy-deficient groups. It was also significantly lower in the frail/energy-sufficient group than the non-frail/energy-sufficient group. The MNA-SF score was significantly lower in the frail/energy-deficient group than in the other three groups, and it was also significantly lower in the non-frail/energy-deficient and frail/energy-sufficient groups than in the non-frail/energy-sufficient group. The BI and FIM were significantly lower in the frail/energy-deficient group than in the other three groups.

Laboratory data showed that albumin was significantly lower in the frail/energy-deficient group than in the non-frail/energy-sufficient and non-frail/energy-deficient groups and was significantly lower in the non-frail/energy-deficient group than in the non-frail/energy-sufficient group. Hemoglobin was significantly lower in the frail/energy-sufficient and frail/energy-deficient groups than in the non-frail/energy-sufficient group. On the other hand, there were no significant differences in the BNP, CRP, creatinine, or total cholesterol among the four groups.

Supplemental Table S2 presents the characteristics by sex. In both males and females, the non-frail/energy-sufficient group was significantly younger and had significantly higher handgrip strength, MNA-SF scores, and ADLs, demonstrating a trend similar to the overall analysis.

The rehabilitation outcomes for each group are presented in Table 2. At discharge, both the body weight and handgrip strength were significantly lower in the frail/energy-deficient group than in the other three groups. The quadriceps strength was significantly lower in the frail/energy-deficient group than in the non-frail/energy-sufficient and frail/energy-sufficient groups. The MNA-SF score at discharge was significantly lower in the frail/energy-deficient group than in the other three groups. The energy intake was significantly lower in the frail/energy-deficient group than in the non-frail/energy-sufficient and frail/energy-sufficient groups.

The mean change in the FOIS score from admission to discharge in the frail/energy-deficient group was −0.2 ± 1.2—the only decrease among the four groups—and was significantly lower than in the frail/energy-sufficient group. There were no differences among the four groups with respect to changes in handgrip strength, quadriceps strength, or MNA-SF score between admission and discharge. The BI and FIM total scores at discharge were significantly lower in the frail/energy-deficient group than in the other three groups. The length of hospital stay was significantly longer in the frail/energy-deficient group than in the non-frail/energy-sufficient and non-frail/energy-deficient groups. It was also significantly longer in the frail/energy-sufficient group than in the non-frail/energy-sufficient group.

Supplemental Table S3 presents the rehabilitation outcomes by sex. In both males and females, the FIM in the frail/energy-deficient group was significantly lower than in the other three groups. Additionally, the FIM efficiency in both the frail/energy-sufficient and frail/energy-deficient groups was significantly lower than in the non-frail/energy-sufficient group. For males, the RE for the non-frail/energy-sufficient, non-frail/energy-deficient, frail/energy-sufficient, and frail/energy-deficient groups were 80.7 [70.5–91.2], 67.6 [58.1–85.0], 56.3 [42.1–70.0], and 50.6 [12.2–68.4], respectively. For females, the RE for the non-frail/energy-sufficient, non-frail/energy-deficient, frail/energy-sufficient, and frail/energy-deficient groups were 76.9 [58.1–90.6], 71.1 [50.2–87.7], 62.1 [40.3–81.1], and 54.4 [37.5–69.5], respectively (Supplemental Figure S2). In both the males and females, the RE in the frail/energy-deficient group was significantly lower than in the other three groups, and the RE in the frail/energy-sufficient group was significantly lower than in the non-frail/energy-sufficient group.

**Figure 2:**
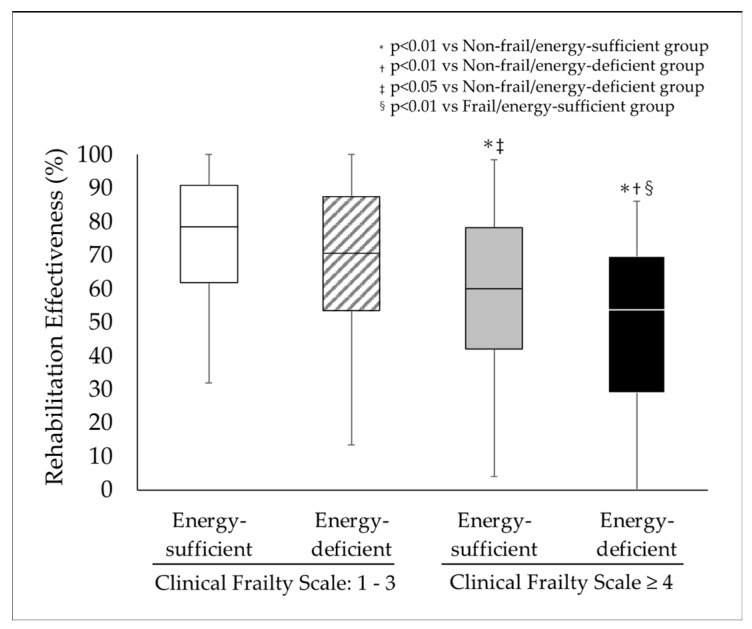
Comparison of rehabilitation effectiveness among groups classified based on the Clinical Frailty Scale and energy intake.

The RE scores for the non-frail/energy-sufficient, non-frail/energy-deficient, frail/energy-sufficient, and frail/energy-deficient groups were 78.5 [61.8–90.7], 70.6 [53.4–87.4], 59.9 [41.9–78.1], and 53.6 [41.9–78.1], respectively (Figure 2). RE was also significantly lower in the frail/and energy-deficient group than in the other three groups and was significantly lower in the frail/energy-sufficient group than in the non-frail/energy-sufficient and non-frail/energy-deficient groups. Although there were no differences in the FIM gain among the four groups, the length of stay was shorter in the non-frail groups. Consequently, the FIM efficiencies in the frail/energy-sufficient and frail/energy-deficient groups were significantly lower than in the non-frail/energy-sufficient and non-frail/energy-deficient group.

**Figure 3:**
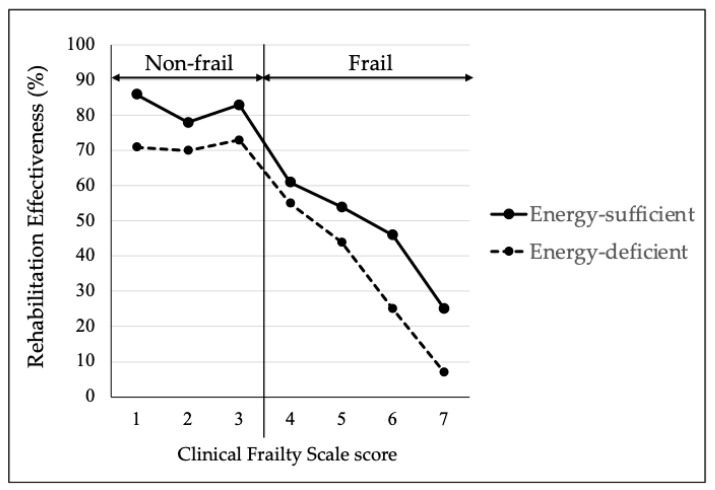
Rehabilitation effectiveness of the energy-sufficient and energy-deficient groups according to the CFS score.

Figure 3 shows the RE for the energy-sufficient and energy-deficient groups based on the CFS. In 465 patients classified as frail, an interaction between the CFS score and sufficient or insufficient energy intake was observed (*p* = 0.024). However, this was not observed in the 270 patients classified as non-frail (*p* = 0.667).

Supplemental Figure S3 presents the rehabilitation effectiveness of the energy-sufficient and energy-deficient groups according to the CFS score by sex. In the males, no interaction was observed between the CFS score and energy sufficiency or deficiency in the overall population, frail group, or non-frail group. However, in the females, an interaction between the CFS and sufficient or insufficient energy intake was observed among the patients classified as frail (*p* = 0.045).

The univariate regression analysis revealed that the age, CFS score, energy intake ratio, handgrip strength, FOIS score, MNA-SF score, BNP, creatinine, hemoglobin, CRP, and total cholesterol were significantly associated with RE. Furthermore, the multivariate regression analysis confirmed that the age, male sex, CFS score, energy intake ratio, handgrip strength, FOIS score, MNA-SF score, BNP, and creatinine were independently and significantly associated with RE (Table 3).

Supplemental Table S4 presents the univariate linear regression analysis and multiple linear regression analysis of rehabilitation effectiveness by sex. In the males, the multivariate regression analysis confirmed that the CFS score, energy intake ratio, handgrip strength, FOIS score, and CRP were independently and significantly associated with RE. In females, the multivariate regression analysis confirmed that the age, CFS score, energy intake ratio, handgrip strength, FOIS score, MNA-SF score, and hemoglobin were independently and significantly associated with RE.

## 4. Discussion

In this study, we investigated the effect of pre-admission CFS scores and the energy intake ratio on the efficiency of ADL improvement in patients with musculoskeletal disorders in convalescent rehabilitation wards. We showed that the frail/energy-deficient group had a lower RE than the other three groups. In addition, the multivariate analysis confirmed that the CFS score and energy intake ratio were significantly and independently associated with the RE after adjusting the age, sex, and potential confounders. The effect of energy deficiency on the RE was more pronounced in the frail groups than in the non-frail groups. This suggests that patients with musculoskeletal disorders who are frail and have a low dietary intake show less efficient improvement in their ADLs and that the effect of low dietary intake is particularly pronounced in frail patients.

From a physiological perspective, frailty is characterized by a reduced muscle mass and strength, decreased metabolic efficiency, and increased systemic inflammation, all of which are strongly associated with a decline in physical function [5,37,38]. Frailty is also considered a pre-disability state and is associated with various negative health outcomes, including falls, hospitalization, institutionalization, fracture, disability, dementia, lower quality of life, and mortality [39,40,41]. Japan’s population is aging rapidly, and the numbers of individuals classified as pre-frail or frail have been increasing. According to a systematic review and meta-analysis, the prevalence of frailty, pre-frailty, and robustness based on the Fried criteria in 11,940 community-dwelling Japanese individuals aged 65 years or older was 7.4%, 48.1%, and 44.4%, respectively [42]. Thus, frailty increases with aging and is reportedly increasing in rehabilitation wards, with adverse effects on physical function outcomes [43]. The prevalence of frailty, as defined by the CFS in the rehabilitation wards analyzed in the present study, was 63%, which may be attributable to the fact that our study population consisted of patients with musculoskeletal disorders who were of a higher average age than the general population. In the frail groups, RE, an indicator of rehabilitation outcomes, was significantly lower than in the non-frail groups, which was consistent with earlier studies [43]. Interestingly, although the length of hospitalization was longer in the frail groups, the increases in the FIM and BI scores were nearly the same as in the non-frail groups. This indicates that although frailty is considered to be a pre-disability state, it can be reversed to some extent through rehabilitation, despite taking a longer time. These results therefore suggest that rehabilitation is effective in restoring physical function, even in frail elderly patients with bone and musculoskeletal disorders.

We investigated the effect of the energy intake ratio on the efficiency of ADL improvement in patients with musculoskeletal disorders. In general, low energy intake is a significant risk factor for complications and mortality in hospitalized patients [44]. Therefore, addressing energy intake in hospitalized patients could be important for functional recovery in musculoskeletal disorders. In this study, a decline in energy intake was seen in 30% of the non-frail patients and half of the frail patients. In the energy-deficient groups, a lower albumin value, MNA-SF score, and grip strength were observed at the time of admission than in the non-frail/energy-sufficient group, and the lower MNA-SF score and grip strength persisted to the time of discharge. Regarding rehabilitation outcomes, the BI and total FIM scores at admission were lower in the energy-deficient groups than in the energy-sufficient groups, and the lower total FIM scores persisted to the time of discharge. Previously, Inoue et al. reported that the baseline FIM score was lower in an energy-deficient group than an energy-sufficient group in patients with hip fractures in an acute care hospital [45], which was consistent with our study. They also showed that the FIM gain was significantly higher in the energy-sufficient group (37 scores vs. 20 scores). By contrast, in our study, there was no significant difference in the FIM gain between the energy-sufficient and energy-deficient groups. One possible explanation for the discrepancy between our findings and those of earlier studies is that the FIM gain in our study was 39 (range: 24–43) points, which is higher than that reported in previous studies (23.8 ± 15.0 points). This suggests a potential ceiling effect. After adjusting for this ceiling effect using RE, we found that the frail patients exhibited a significantly lower RE and that the energy-deficient groups had significantly lower FIM scores at discharge than the energy-sufficient groups.

We also examined the interaction between dietary intake and frailty on RE. As shown in Figure 3, the effect of dietary intake deficiency on RE was small in the non-frail patients, whereas the effect of dietary intake deficiency on RE was greater in the patients who were frail. There was an interaction between dietary intake and frailty (*p*-interaction = 0.024). The decreased and/or delayed functional recovery from energy deficiency may have been caused by a decline in skeletal muscle mass and function. In fact, the energy-deficient groups had lower nutritional statuses and lower handgrip strengths in this study. This effect of inadequate energy intake on RE was more prominent in the patients in the frail/energy-deficient group. The exact reason why energy intake deficiency has a stronger effect on rehabilitation outcomes in frail patients than those who are not frail is unclear; however, there are several possible reasons. Increased levels of cytokines such as CRP, IL-1β, IL-6, and TNF-α have been reported in frailty [46]. The inflammatory state induced by increased cytokines levels enhances catabolic responses and leads to the breakdown of skeletal muscle proteins, which contributes to muscle dysfunction. Because frail patients have less muscle mass and strength, inadequate energy intake is likely to lead to further declines in physical function. Thus, a potential molecular mechanism may be that pro-inflammatory cytokines directly affect frailty by promoting protein degradation and/or indirectly affect frailty via important metabolic pathways. Further study will be needed to elucidate the mechanisms by which low energy intake affects the decline in physical function in frail patients.

Interestingly, BNP (pg/mL), a marker of heart failure, was significantly associated with RE in this study. This finding suggests that heart failure may influence rehabilitation outcomes. We used plasma BNP levels as a surrogate marker for the diagnosis of heart failure. BNP is a hormone produced and secreted by the heart [47] and is known to be increased in left ventricular hypertrophy [48], acute myocardial infarction [49], coronary artery disease [50], and renal failure [51] as well as in heart failure. We previously showed that rehabilitation is attenuated in hip fracture patients diagnosed with heart failure, based on plasma BNP ≥ 100 pg/mL, and that nutritional disorders and heart failure are additively associated with the attenuated rehabilitation effect [22,35]. On the other hand, creatinine (mg/dL) was positively correlated with RE in this study, meaning that an increase in creatinine leads to improved rehabilitation outcomes. Creatinine is a marker for renal failure, and a previous study showed that it is negatively correlated with rehabilitation outcomes [52]. In the present study, the average Cre values in all groups were 0.8–0.9, which was within the normal upper limit. Considering that creatinine is of muscle origin [53], it is suggested that creatinine may be positively correlated with rehabilitation outcomes in patients whose rehabilitation improves nutritional indices and increases muscle mass. The fact that, in this study, male gender was positively correlated with improvement in RE when all patients were examined, whereas creatinine did not correlate with improvement in RE when patients were examined by gender, supports the above idea.

This study has several limitations. First, as a retrospective study conducted at a single hospital, the generalizability of the results may be limited. Additionally, since this study was conducted in a convalescent rehabilitation ward, patients with more severe conditions may not have been included, which could affect the applicability of the findings. Multicenter prospective cohort studies are needed to overcome these limitations. Second, we investigated energy intake ratios only at admission. However, the energy intake at later times after admission may be equally or more important. Additionally, energy intake was assessed using a visual estimation method, which may introduce subjective bias. Third, we did not investigate the intake of each nutrient in the present study. Consequently, the degrees to which different nutrients among proteins, carbohydrates, and fat contribute to RE remain unknown. Because of the retrospective observational design of this study, the causal relationship between baseline malnutrition and outcomes is unclear.

However, a strength of this study is that we were able to determine the amount of energy taken in by patients based on their BEEs, calculated using the Harris–Benedict equation, and the activity coefficient, established through multidisciplinary discussions. This approach enabled us to assess each patient’s energy intake rate relative to their individualized appropriate energy intake. We believe that the assessment of frailty, combined with appropriate nutritional management and a tailored rehabilitation program, including early gait training [54] and muscle strength training [55], based on patients’ condition, contributes to more effective rehabilitation outcomes and the improvement of patients’ ADLs.

Dietary assessment is a cornerstone of nutrition-related research and plays an important role in managing chronic diseases such as diabetes mellitus. However, conventional dietary assessment methods are time-consuming, labor-driven, and inaccurate. For future research directions, recent development of artificial intelligence (AI)-assisted dietary assessment tools can provide real-time objective and more accurate data by allowing food recognition; food classification; and food volume, food weight, and nutrient estimation automatically compared to traditional dietary assessment methods [56,57,58]. Furthermore, these tools are hassle-free, time-efficient, and user-friendly, and provide reasonably accurate data to enable clinicians to provide personalized nutrition [59]. Thus, AI-assisted dietary assessment tools will probably improve the quality of nutritional care [60] and could be applied to the nutritional assessment of patients in rehabilitation wards. Future prospective randomized controlled studies using an AI-assisted dietary assessment tool will be necessarily to determine whether it is effective in improving ADL outcomes in patients with frailty in rehabilitation ward.

## 5. Conclusions

In conclusion, our data suggest that CFS scores and energy intake ratios independently influence the efficiency of ADL improvement in patients with musculoskeletal disorders admitted to convalescent rehabilitation wards.

## Figures and Tables

**Table 1:** Demographic and clinical data for each group based on Clinical Frailty Scale scores and energy intake classification at admission.

	Total	Non-Frail/Energy-Sufficient Group	Non-Frail/Energy-Deficient Group	Frail/Energy-Sufficient Group	Frail/Energy-Deficient Group	*p*
*n*	735	184	86	230	235	<0.001
Age, year	81 ± 10	77 ± 12	81 ± 10 *	81 ± 9 *	85 ± 7 **^†¶^	<0.001
Male, *n* (%)	202 (27.5)	62 (33.7)	20 (23.3)	77 (33.5)	43 (18.3) *^§^	<0.001
CFS, score	3.8 ± 1.2	2.6 ± 0.7	2.8 ± 0.6	4.4 ± 0.8 *	4.5 ± 0.9 *^†^	<0.001
Energy intake ratio, (%)	84.9± 21.3	100 ± 0	72.3 ± 19.9 *	100 ± 0 ^†^	63.7 ± 19.1 *^†§^	<0.001
Height, m	1.52 ± 0.1	1.54 ± 0.1	1.52 ± 0.1	1.52 ± 0.1	1.49 ± 0.1 *^‡¶^	<0.001
Weight, kg	49.0 ± 10.3	52.5 ± 10.1	50.5 ± 10.2	49.1 ± 10.6 *	45.6 ± 9.1 *^†§^	<0.001
Body mass index kg/m^2^	21.3 ± 3.6	22.1 ± 3.6	21.7 ± 3.3	21.1 ± 3.6	20.6 ± 3.4 *	<0.001
Comorbidity *n* (%)						
Hypertension	446 (60.7)	114 (60.2)	51 (59.3)	132 (57.4)	149 (63.4)	0.580
Diabetes mellitus	162 (22.0)	51 (27.7)	14 (16.3)	58 (25.2)	39 (16.6) *	0.014
Dyslipidemia	162 (22.0)	42 (22.8)	15 (17.4)	51 (22.2)	54 (23.0)	0.728
Atrial fibrillation	55 (7.5)	12 (6.5)	8 (9.3)	17 (7.4)	18 (7.7)	0.884
Handgrip strength, kg	15.5 ± 7.9	19.0 ± 9.1	16.1 ± 7.4 **	15.2 ± 7.7 *	12.6 ± 5.9 *^†§^	<0.001
Quadriceps strength, kg	12.3 ± 6.8	13.7 ± 7.4	12.8 ± 6.7	12.4 ± 6.8	10.7 ± 5.8 *	0.001
Thigh circumference, cm	35.9 ± 5.1	37.7 ± 4.8	36.7 ± 6.1	35.5 ± 5.2 *	34.6 ± 4.5 *^‡^	<0.001
Calf circumference, cm	28.7 ± 3.8	30.3 ± 3.3	28.8 ± 4.4 **	28.5 ± 3.9 *	27.6 ± 3.2 *	<0.001
FOIS	6.4 ± 1.0	6.8 ± 0.5	6.5 ± 0.8	6.3 ± 1.2 *	6.2 ± 1.0 *^‡^	<0.001
Barthel Index, score	50 (35–70)	70 (55–80)	58 (40–70) *	50 (35–60) *	40 (25–50) *^†§^	<0.001
FIM, score						
Motor	37 (25–47)	47 (39–56)	40 (27–48) *	36 (24–45) *	30 (22–37) *^†§^	<0.001
Cognitive	23 (18–27)	26 (22–31)	25 (20–29) **	22 (17–27) *	21 (16–25) *^†^	<0.001
Total	59 (45–75)	74 (63–86)	66 (48–77) *	57 (42–70) *	49 (40–61) *^†§^	<0.001
MNA-SF	6.7 ± 2.5	7.8 ± 2.1	6.6 ± 2.3 *	6.7 ± 2.6 *	5.7 ± 2.5 *^‡§^	<0.001
GNRI	90.6 ± 11.1	95.5 ± 11.1	91.1± 11.1	89.1 ± 11.4 *	88.2 ± 9.5 *	<0.001
Energy intake, kcal	1365 ± 376	1619 ± 262	1151 ± 313 *	1554 ± 202	1059 ± 343 *^†§^	<0.001
Medication, *n*	5 ± 3	5 ± 3	5 ± 3	6 ± 3	6 ± 3	0.487
BNP, pg/mL	41 (28–86)	31 (16–73)	43 (24–83)	43 (25–86)	48 (22–99)	0.929
Albumin, g/dL	3.5 ± 0.5	3.8 ± 0.4	3.5 ± 0.5 *	3.4 ± 0.5	3.3 ± 0.5 *^‡^	<0.001
CRP, mg/dL	0.0 (0.0–1.0)	0.0 (0.0–0.9)	0.0 (0.0–1.0)	0.0 (0.0–1.0)	0.2 (0.0–1.0)	0.123
Creatinine, mg/dL	0.8 ± 0.4	0.8 ± 0.4	0.9 ± 0.4	0.8 ± 0.4	0.8 ± 0.4	0.903
Total cholesterol, mg/dL	185 ± 40	188 ± 44	189 ± 38	180 ± 36	185 ± 40	0.111
Hemoglobin, g/dL	11.5 ± 1.6	11.9 ± 1.6	11.6 ± 1.4	11.4 ± 1.6 *	11.4 ± 1.6 *	0.006

* *p* < 0.01 vs. the non-frail/energy-sufficient group. ** *p* < 0.05 vs. the non-frail/energy-sufficient group. † *p* < 0.01 vs. non-frail/energy-shortage group. ‡ *p* < 0.05 vs. the non-frail and energy-deficient group. § *p* < 0.01 vs. the frail/energy-sufficient group. ¶ *p* < 0.05 vs. frail/energy-sufficient group. BNP: Brain (B-type) Natriuretic Peptide; CFS; Clinical Frailty Scale; CRP; *C*-reactive protein; FIM: Functional Independence Measure; FOIS: Functional Oral Intake Scale; GNRI: Geriatric Nutritional Risk Index; MNA-SF: Mini Nutritional Assessment-Short Form.

**Table 2:** Rehabilitation outcomes for each group based on Clinical Frailty Scale scores and energy intake classification at discharge.

	Total	Non-Frail/Energy-Sufficient Group	Non-Frail/Energy-Deficient Group	Frail/Energy-Sufficient Group	Frail/Energy-Deficient Group	*p*
*n*	735	184	86	230	235	
Energy intake ratio, (%)	92.9 ± 13.3	99.9 ± 2.2	86.0 ± 15.0 *	96.3 ± 11.1 *^†^	86.9 ± 15.6 *^§^	<0.001
Weight, kg	48.7 ± 10.0	52.5 ± 9.8	49.9 ± 9.8 *	48.7 ± 10.4 *^†^	45.2 ± 8.7 *^†§^	<0.001
Body mass index kg/m^2^	21.1 ± 3.4	22.1 ± 3.5	21.5 ± 3.2 *	20.9 ± 3.5 *	20.4 ± 3.3 *	<0.001
Handgrip strength, kg	15.9 ± 7.8	19.3 ± 8.8	15.8 ± 8.0 *	15.9 ± 7.6 *	13.1 ± 5.8 *^‡§^	<0.001
Quadriceps strength, kg	14.4 ± 7.1	16.4 ± 7.3	14.2 ± 7.1	14.5 ± 7.0	12.7 ± 6.5 *^¶^	<0.001
Thigh circumference, cm	36.1± 4.8	37.8 ± 4.4	37.1 ± 4.8	35.8 ± 5.1 *	34.7 ± 4.5 *^†^	<0.001
Calf circumference, cm	29.3 ± 3.7	30.8 ± 3.4	29.8 ± 3.8	29.1 ± 3.7 *	28.0 ± 3.2 *^†§^	<0.001
FOIS	6.4 ± 1.1	6.8 ± 0.5	6.5 ± 1.0	6.3 ± 1.2 *	6.0 ± 1.3 *^†^	<0.001
MNA-SF	9.7 ± 2.7	11.0 ± 2.1	10.1 ± 2.2 **	9.4 ± 2.7 *	8.7 ± 2.7 *^¶^	<0.001
GNRI	91.0 ± 10.5	95.8 ± 10.2	91.1 ± 10.7	89.9 ± 10.3 *	88.2 ± 9.6 *	<0.001
Energy intake, kcal	1509 ± 309	1653 ± 278	1432 ± 316 *	1555 ± 264 *^†^	1378 ± 312 *^§^	<0.001
Barthel Index, score	90 (70–100)	100 (90–100)	90 (85–100)	90 (70–100) *	80 (55–90) *^†§^	<0.001
Changes during hospitalization						
Change in body weight	−0.4 ± 2.4	0.0 ± 2.2	−0.6 ± 2.1	−0.4 ± 2.8	−0.4 ± 2.3	0.354
Change in handgrip strength	0.6 ± 3.9	0.6 ± 3.2	−0.1 ± 5.0	0.8 ± 3.8	0.6 ± 4.0	0.683
Change in quadriceps strength	2.3 ± 4.8	2.7 ± 5.0	1.4 ± 4.5	2.5 ± 5.1	2.1 ± 4.2	0.999
Change in FOIS	0.0 ± 0.8	0.0 ± 0.3	0.0 ± 0.9	0.0 ± 0.7	−0.2 ± 1.2^¶^	<0.001
Change in MNA-SF	3.0 ± 2.6	3.3 ± 2.3	3.4 ± 2.5	2.7 ± 2.6	3.0 ± 2.8	0.139
Change in GNRI	0.4 ± 4.7	0.4 ± 5.0	0.1 ± 5.0	0.8 ± 4.4	0.0 ± 4.8	0.687
FIM, score						
Motor	75 (62–84)	85 (77–88)	80 (67–85) **	74 (61–83) *	67 (52–77) *^†§^	<0.001
Cognitive	28 (22–33)	33 (28–35)	30 (23–35)	26 (19–32) *^†^	25 (20–30) *^†^	<0.001
Total	103 (84–117)	117 (106–122)	108 (91–119) **	98 (80–115) *^‡^	92 (74–105) *^†§^	<0.001
Barthel Index gain, score	30 (20–45)	25 (15–40)	30 (20–45)	30 (20 -45)	35(20–50)	0.067
FIM gain, score						
Motor	35(24–43)	35 (25–42)	37(29–44)	34(25–43)	35(21–43)	0.096
Cognitive	3 (0–6)	3 (1–6)	4 (0–7)	3 (0–6)	3 (0–6)	0.921
Total	39 (27–48)	38 (28–46)	41 (31–49)	38 (26–47)	40 (23–49)	0.116
Length of hospital stay, day	78 (56–87)	62 (44–81)	74 (52–87)	79 (59–88) *	84 (64–88) *^‡^	<0.001
FIM efficiency, score/day	0.54 (0.37–0.74)	0.61 (0.44–0.83)	0.60 (0.42–0.87)	0.51 (0.36–0.70) *^†^	0.51 (0.28–0.64)*^†^	0.002

* *p* < 0.01 vs. the non-frail/energy-sufficient group. ** *p* < 0.05 vs. the non-frail/energy-sufficient group. † *p* < 0.01 vs. the non-frail/energy-deficient group. ‡ *p* < 0.05 vs. the non-frail/energy-deficient group. § *p* < 0.01 vs. the frail/energy-sufficient group. ¶ *p* < 0.05 vs. frail/energy-sufficient group. FIM: Functional Independence Measure; FOIS: Functional Oral Intake Scale; MNA-SF: Mini Nutritional Assessment-Short Form.

**Table 3:** Univariate linear regression analysis and multiple linear regression analysis of rehabilitation effectiveness.

	Univariate Linear Regression Analysis					Multiple Linear Regression Analysis				
	B	β	*p*	95%CI		B	β	*p*	95%CI	
				Lower	Higher				Lower	Higher
						11.748		0.419	−16.809	40.304
Age	−0.961	−0.345	<0.001	−1.150	−0.771	−0.361	−0.134	<0.001	−0.552	−0.169
Male	1.990	0.033	0.370	−2.366	6.345	−10.949	−0.184	<0.001	−15.621	−6.277
CFS	−7.989	−0.346	<0.001	−9.559	−6.420	−3.047	−0.131	<0.001	−4.615	−1.480
Energy intake ratio	0.471	0.357	<0.001	0.387	0.556	0.281	0.220	<0.001	0.195	0.368
Handgrip strength	1.318	0.395	<0.001	1.085	1.551	0.751	0.227	<0.001	0.467	1.036
FOIS	10.915	0.402	<0.001	9.044	12.786	5.319	0.192	<0.001	3.371	7.267
MNA-SF	4.457	0.423	<0.001	3.761	5.153	1.268	0.120	0.001	0.503	2.033
BNP	−0.027	−0.108	0.004	−0.045	−0.009	−0.017	−0.073	0.041	−0.033	−0.001
Creatinine	5.945	0.083	0.024	0.769	11.121	9.687	0.141	<0.001	4.728	14.645
Hemoglobin	3.315	0.197	<0.001	2.115	4.514	0.776	0.045	0.201	−0.415	1.966
CRP	−1.940	−0.108	0.003	−3.231	−0.648	−0.088	−0.005	0.877	−1.207	1.031
Total cholesterol	0.052	0.076	0.040	0.002	0.101	−0.009	−0.013	0.706	−0.054	0.037

BNP: Brain (B-type) Natriuretic Peptide; CFS: Clinical Frailty Scale; CRP: *C*-reactive protein; FOIS: Functional Oral Intake Scale; MNA-SF: Mini Nutritional Assessment-Short Form.

## Data Availability

The original contributions presented in this study are included in the article and Supplementary Materials. Further inquiries can be directed to the corresponding author.

## Supplementary Materials

The following supporting information can be downloaded at https://www.mdpi.com/article/10.3390/nu17081334/s1: Figure S1: CLINICAL FRAILTY SCALE; Figure S2: Comparison of rehabilitation effectiveness among groups classified based on the Clinical Frailty Scale and energy intake by sex; Figure S3: Rehabilitation effectiveness of the energy-sufficient and energy-deficient groups according to CFS by sex; Table S1: Activity factors and rehabilitation programs; Table S2: Demographic and clinical data for each group by sex based on Clinical Frailty Scale Scores and energy intake classification at admission; Table S3: Rehabilitation outcomes for each group by sex based on Clinical Frailty Scale scores and energy intake classification at discharge; Table S4: Univariate linear regression analysis and multiple linear regression analysis of rehabilitation effectiveness by sex.

## Abbreviations

The following abbreviations are used in this manuscript:

ADL	activities of daily living
ANCOVA	analysis of covariance
ANOVA	analysis for a one-way analysis of variance
BEE	basal energy expenditure
BI	Barthel Index
BMI	body mass index
BNP	Brain (B-type) Natriuretic Peptide
CFS	Clinical Frailty Scale
CRP	*C*-reactive protein
FIM	Functional Independence Measure
FOIS	Functional Oral Intake Scale
GNRI	Geriatric Nutritional Risk Index
HBE	Harris–Benedict equation
MNA-SF	Mini Nutritional Assessment-Short Form
RE	rehabilitation effectiveness
