# Supplementary material for: Frailty and Energy Intake Deficiency Reduce the Efficiency of Activities of Daily Living in Patients with Musculoskeletal Disorders: A Retrospective Cohort Study
Source: Nutrients. 2025 Apr 12;17(8):1334. doi: 10.3390/nu17081334 (PMC12030590; doi:10.3390/nu17081334)
Supplement: Supplementary file 1 [file nutrients-17-01334-s001.zip › nutrients-3524404-supplementary.pdf]

Supplemental Figure S1. CLINICAL FRAILTY SCALE

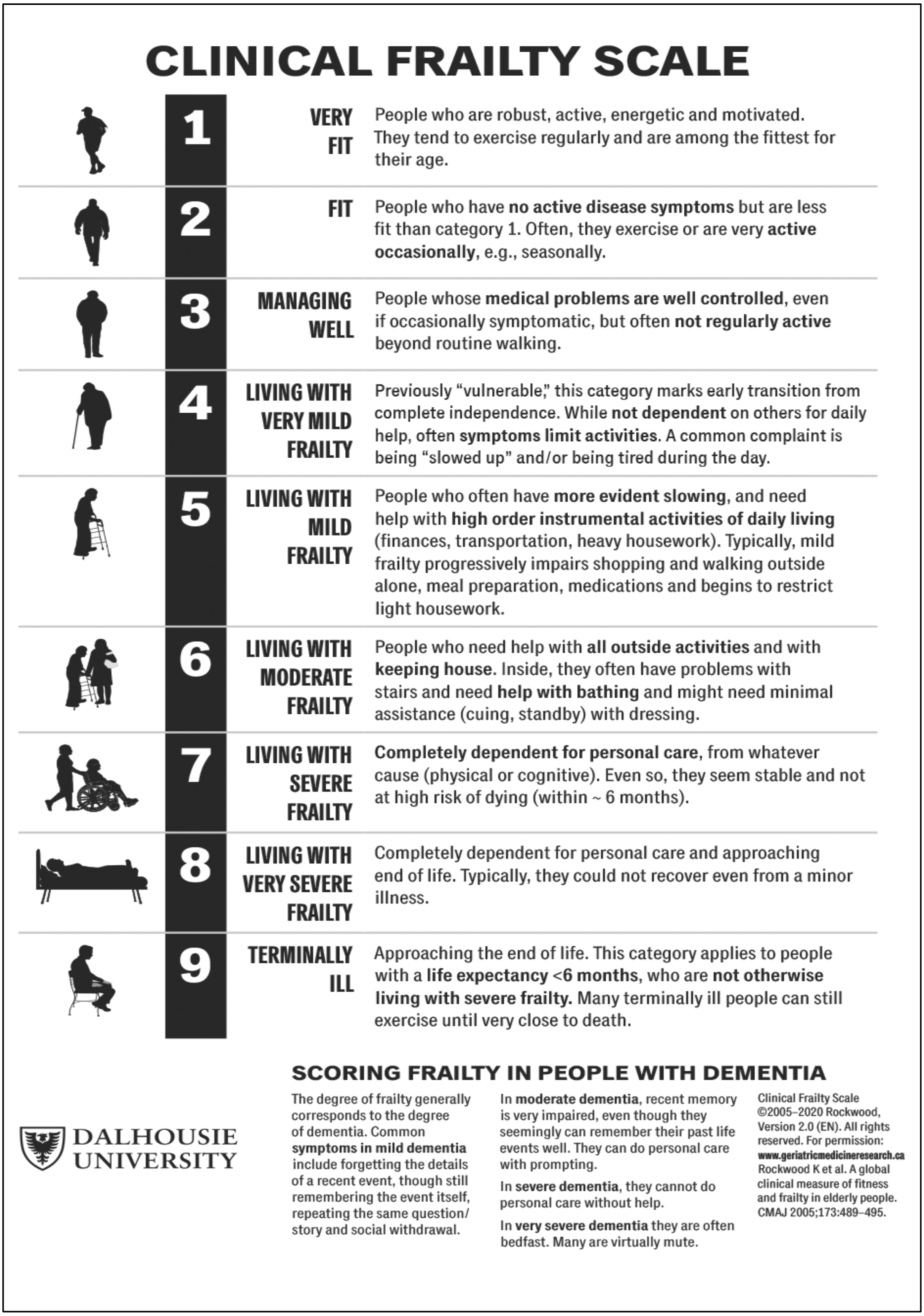

CLINICAL FRAILTY SCALE

1	VERY FIT	People who are robust, active, energetic and motivated. They tend to exercise regularly and are among the fittest for their age.
2	FIT	People who have **no active disease symptoms** but are less fit than category 1. Often, they exercise or are very **active occasionally**, e.g., seasonally.
3	MANAGING WELL	People whose **medical problems are well controlled**, even if occasionally symptomatic, but often **not regularly active** beyond routine walking.
4	LIVING WITH VERY MILD FRAILTY	Previously “vulnerable,” this category marks early transition from complete independence. While **not dependent** on others for daily help, often **symptoms limit activities**. A common complaint is being “slowed up” and/or being tired during the day.
5	LIVING WITH MILD FRAILTY	People who often have **more evident slowing**, and need help with **high order instrumental activities of daily living** (finances, transportation, heavy housework). Typically, mild frailty progressively impairs shopping and walking outside alone, meal preparation, medications and begins to restrict light housework.
6	LIVING WITH MODERATE FRAILTY	People who need help with **all outside activities** and with **keeping house**. Inside, they often have problems with stairs and need **help with bathing** and might need minimal assistance (cuing, standby) with dressing.
7	LIVING WITH SEVERE FRAILTY	**Completely dependent for personal care**, from whatever cause (physical or cognitive). Even so, they seem stable and not at high risk of dying (within ~ 6 months).
8	LIVING WITH VERY SEVERE FRAILTY	Completely dependent for personal care and approaching end of life. Typically, they could not recover even from a minor illness.
9	TERMINALLY ILL	Approaching the end of life. This category applies to people with a **life expectancy <6 months**, who are **not otherwise living with severe frailty.** Many terminally ill people can still exercise until very close to death.

SCORING FRAILTY IN PEOPLE WITH DEMENTIA

The degree of frailty generally corresponds to the degree of dementia. Common **symptoms in mild dementia** include forgetting the details of a recent event, though still remembering the event itself, repeating the same question/story and social withdrawal.

In **moderate dementia**, recent memory is very impaired, even though they seemingly can remember their past life events well. They can do personal care with prompting.

In **severe dementia**, they cannot do personal care without help.

In **very severe dementia** they are often bedfast. Many are virtually mute.

Clinical Frailty Scale ©2005–2020 Rockwood, Version 2.0 (EN). All rights reserved. For permission: www.geriatricmedicineresearch.ca

Rockwood K et al. A global clinical measure of fitness and frailty in elderly people. CMAJ 2005;173:489–495.

DALHOUSIE UNIVERSITY

Supplemental Figure S2. Comparison of Rehabilitation Effectiveness Among Groups Classified based on the Clinical Frailty Scale and Energy Intake by sex.

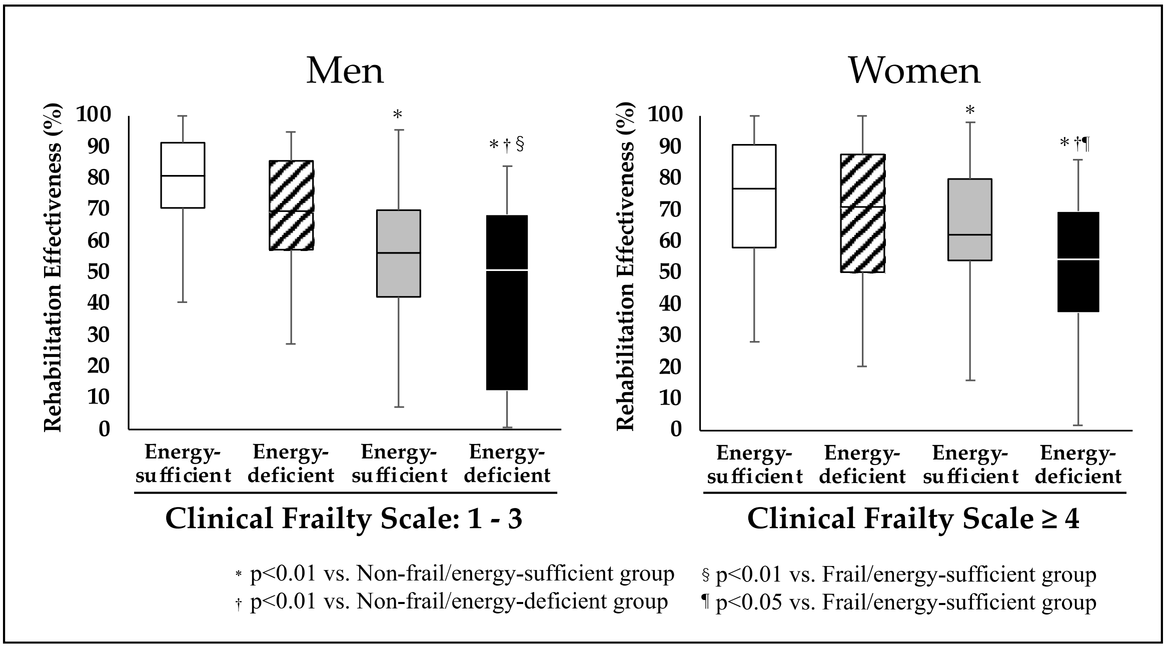

Supplemental Figure S3. Rehabilitation Effectiveness of the energy-sufficient and energy-deficient groups according to CFS by sex.

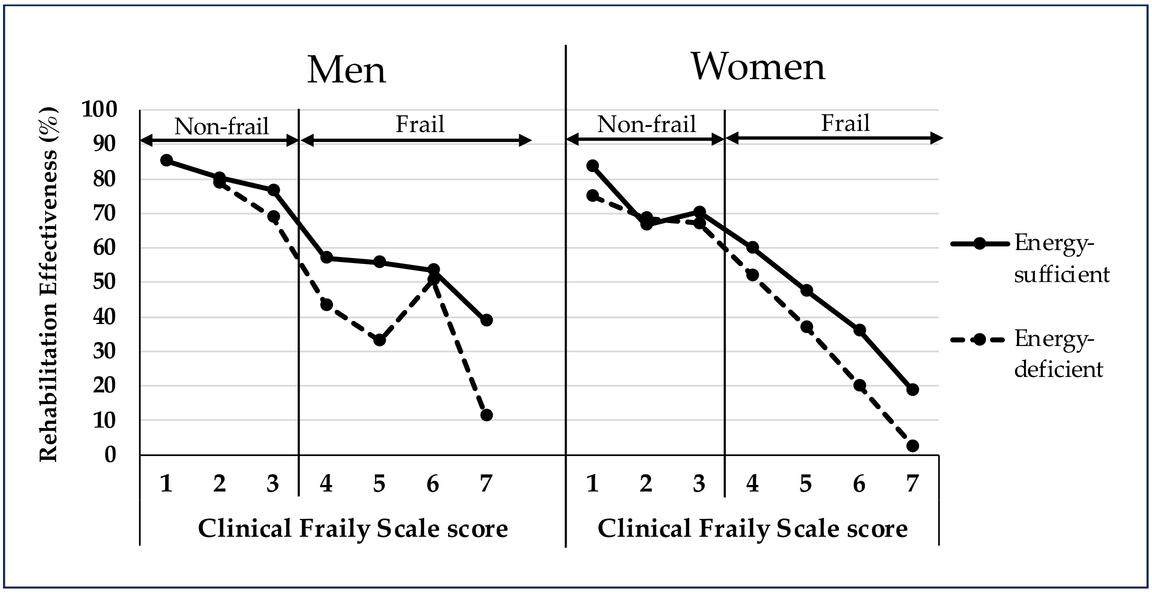

Supplemental Table S1. Activity Factors and Rehabilitation Programs

Activity Factor	Rehabilitation setting	Rehabilitation therapy
1.0	Bed rest<br>(JCS scores ranging from two to three digits)	Stretching<br>ROM exercises
1.1	Bed rest<br>(JCS single-digit scores)	Stretching<br>ROM exercises<br>Muscle strengthening exercises
1.2	Bedside rehabilitation	Stretching<br>ROM exercises<br>Muscle strengthening exercises<br>Sitting training
1.3	Out-of-bed activities<br>Approximately 20-minute rehabilitation therapy	ROM exercises<br>Muscle strengthening exercises<br>Sitting training<br>Standing training<br>Balance training
1.4	Rehabilitation in the therapy room<br>Approximately 60-minute rehabilitation therapy	ROM exercises<br>Muscle strengthening exercises<br>Sitting training<br>Standing training<br>Balance training<br>Walking training
1.5	Rehabilitation in the therapy room<br>Approximately 120-minute rehabilitation therapy	ROM exercises<br>Muscle strengthening exercises<br>Sitting training<br>Standing training<br>Balance training<br>Walking training<br>ADL training appropriate for the living environment

ADL; activities of daily living, JCS; Japan Coma Scale, ROM; range of motion

Supplemental Table S2. Demographic and Clinical Data for Each Group by Sex Based on Clinical Frailty Scale Scores and Energy Intake Classification at Admission

Men	Total	Non-frail/energy-sufficient group	Non-frail/energy-deficient group	Frail/energy-sufficient group	Frail/energy-deficient group	p
n	202	62	20	77	43	
Age, year	78 ± 12	73 ± 15	78 ± 15 *	80 ± 10 *	83 ± 7 *†¶	<0.001
CFS, score	3.8 ± 1.1	2.7 ± 0.6	2.9 ± 0.2	4.4 ± 0.7 *†	4.6 ± 0.2 *†	<0.001
Energy intake ratio, (%)	88.4 ± 20.2	100 ± 0	71.3 ± 19.8	100 ± 0 †	59.6 ± 18.0 *†§	<0.001
Height, m	1.62 ± 0.1	1.63 ± 0.1	1.64 ± 0.1	1.61 ± 0.1	1.59 ± 0.1 **	0.031
Weight, kg	55.3 ± 11.2	57.1 ± 11.7	60.0 ± 10.8	55.5 ± 10.8	50.1 ± 9.8 *†	0.002
Body Mass Index kg/m^2	21.1 ± 3.4	21.4 ± 3.4	22.1 ± 3.2	21.3 ± 3.5	19.8 ± 3.2 ‡	0.039
Comorbidity n (%)						
Hypertension	119 (58.9)	36 (58.1)	12 (60.0)	48 (62.3)	23 (54.5)	0.820
Diabetes mellitus	48 (23.8)	16 (25.8)	3 (15.0)	18 (23.4)	11 (25.6)	0.783
Dyslipidemia	37 (18.3)	11 (17.7)	3 (15.0)	14 (18.2)	9 (20.9)	0.949
Atrial fibrillation	21 (10.4)	6 (9.7)	4 (20.0)	6 (7.8)	5 (11.6)	0.450
Handgrip strength, kg	21.8 ± 9.3	27.3 ± 9.7	23.1 ± 9.0	20.3 ± 7.6 *	16.3 ± 7.8 *‡	<0.001
Quadriceps strength, kg	15.2 ± 8.0	16.9 ± 8.5	16.0 ± 10.1	15.4 ± 7.5	11.6 ± 6.1**	0.032
Thigh circumference, cm	36.6 ± 5.3	37.6 ± 5.4	39.4 ± 4.9	36.6 ± 5.2	33.8 ± 4.6 *¶	<0.001
Calf circumference, cm	29.4 ± 4.0	30.8 ± 3.8	29.7 ± 5.6	29.1 ± 3.6	27.7 ± 3.3 *	0.001
FOIS	6.4 ± 1.0	6.8 ± 0.6	6.6 ± 0.8	6.1 ± 1.2 *	6.1 ± 1.0 *	<0.001
Barthel Index, score	50 (35 - 70)	70 (55 - 80)	60 (45 - 65) *	50 (35 - 65) *†	35 (20 - 50) *†§	<0.001
FIM, score						
Motor	37 (26 - 50)	51 (41 - 58)	35 (30 - 48) *	36 (24 - 46) *	26 (20 - 35) *‡§	<0.001
Cognitive	23 (18 - 28)	26 (25 - 33)	24 (20 - 29)	21 (16 - 25) *	20 (14 - 23) *‡	<0.001
Total	59 (45 - 76)	78 (67 - 89)	59 (50 - 75) *	57 (42 - 69) *	47 (37 - 58) *‡§	<0.001
MNA-SF	6.6 ± 2.6	7.8 ± 2.1	6.6 ± 2.2	6.8 ± 2.7	4.8 ± 2.2 *‡§	<0.001
GNRI	91.8 ± 12.1	90.6 ± 10.3	93.0± 11.6	90.8 ± 12.5	86.8 ± 12.1 *	0.001
Energy intake, kcal	1549 ± 413	1784 ± 327	1192 ± 416 *	1683 ± 194 †	1133 ± 400 *§	<0.001
Medication, n	5 ± 3	5 ± 3	6 ± 3	5 ± 3	6 ± 3	0.155
BNP, pg/ml	36 (16 - 80)	33 (14 - 74)	35 (22 - 85)	36 (19 - 79)	36 (19 - 75)	0.537
Albumin, g/dl	3.5 ± 0.6	3.7 ± 0.5	3.4 ± 0.6	3.4 ± 0.5 *	3.3 ± 0.6 *	<0.001
CRP, mg/dl	0.1 (0.0 – 1.0)	0.0 (0.0 – 1.0)	0.1 (0.0 – 1.0)	0.2 (0.0 – 1.0)	0.3 (0.0 – 1.0)	0.665
Creatinine, mg/dl	1.0 ± 0.5	1.0 ± 0.5	1.3 ± 0.8	1.0 ± 0.5	1.0 ± 0.3	0.151
Total cholesterol, mg/dl	172 ± 38	175 ± 40	165 ± 40	174 ± 34	166 ± 41	0.463
Hemoglobin, g/dl	11.9 ± 1.8	11.9 ± 1.8	12.3 ± 1.5	11.8 ± 0.9	12.0 ± 1.8*	0.768

Women	Total	Non-frail/energy-sufficient group	Non-frail/energy-deficient group	Frail/energy-sufficient group	Frail/energy-deficient group	p
n	533	122	66	153	192	
Age, year	82 ± 8	78 ± 10	82 ± 7 **	82 ± 8 *	85 ± 7 *¶	<0.001
CFS, score	3.8 ± 1.2	2.5 ± 0.7	2.7 ± 0.6	4.4 ± 0.8 *†	4.4 ± 0.9 *†	<0.001
Energy intake ratio, (%)	83.9± 21.6	100 ± 0	72.6 ± 19.8 *	100 ± 0 †	64.7 ± 19.3 *†§	<0.001
Height, m	1.48 ± 0.1	1.49 ± 0.1	1.48 ± 0.1	1.48 ± 0.1	1.46 ± 0.1 *	0.002
Weight, kg	46.6 ± 8.9	50.2 ± 8.4	47.6 ± 8.1	45.9 ± 9.0	44.6 ± 8.7 *	<0.001
Body Mass Index kg/m^2	21.3 ± 3.6	22.5 ± 3.7	21.6 ± 3.4	21.0 ± 3.6 *	20.8 ± 3.4 *	<0.001
Comorbidity n (%)						
Hypertension	327 (61.4)	78 (63.9)	39 (60.0)	84 (54.9)	126 (65.6)	0.199
Diabetes mellitus	114 (21.4)	35 (28.7)	11 (16.9)	40 (26.1)	28 (14.6) *	0.007

Supplemental Table S2. Cont.

Women	Total	Non-frail/energy-sufficient group	Non-frail/energy-deficient group	Frail/energy-sufficient group	Frail/energy-deficient group	p
Dyslipidemia	125 (23.5)	31 (25.4)	12 (18.5)	37 (24.2)	45 (23.4)	0.723
Atrial fibrillation	34 (6.4)	6 (4.9)	4 (6.2)	11 (7.2)	13 (6.8)	0.881
Handgrip strength, kg	13.0 ± 5.7	15.1 ± 5.4	14.1 ± 5.4	12.5 ± 6.3 *	11.7 ± 54.9 *‡	<0.001
Quadriceps strength, kg	11.2 ± 5.9	12.2 ± 6.4	11.9 ± 5.2	10.8 ± 5.8	10.5 ± 5.7	0.109
Thigh circumference, cm	35.7 ± 5.0	37.7 ± 4.5	35.7 ± 6.2	35.0 ± 5.1 *	34.8 ± 4.5 *	<0.001
Calf circumference, cm	28.4 ± 3.6	30.0 ± 3.1	28.5 ± 3.9 **	28.2 ± 4.1 *	27.6 ± 3.2 *	<0.001
FOIS	6.4 ± 1.0	6.8 ± 0.5	6.5 ± 0.9	6.3 ± 1.2 *	6.2 ± 1.0 *	<0.001
Barthel Index, score	50 (30 - 65)	65 (50 - 75)	55 (35 - 70) **	50 (35 - 65) *	40 (25 - 50) *†§	<0.001
FIM, score						
Motor	37 (24 - 46)	47 (39 - 53)	41 (27 - 48) *	36 (25 - 45) *	30 (22 - 38) *†§	<0.001
Cognitive	23 (18 - 27)	26 (23 - 31)	25 (20 - 29)	23 (17 - 27) *	21 (17 - 25) *†	<0.001
Total	59 (45 - 73)	74 (63 - 83)	68 (47 - 77) *	57 (44 - 71) *	50 (41 - 63) *†§	<0.001
MNA-SF	6.7 ± 2.5	7.8 ± 2.2	6.7 ± 2.3 **	6.7 ± 2.6 *	5.9 ± 2.5 *¶	<0.001
GNRI	92.5 ± 11.2	99.0 ± 10.8	93.3 ± 10.9 *	91.2 ± 11.5 *	89.1 ± 9.4 *‡	<0.001
Energy intake, kcal	1296 ± 336	1535 ± 168	1139 ± 277 *	1489 ± 172 ‡	1043 ± 328 *‡§	<0.001
Medication, n	6 ± 3	6 ± 3	5 ± 3	6 ± 3	6 ± 3	0.400
BNP, pg/ml	44 (22 - 86)	30 (18 - 69)	45 (25 - 79)	47 (25 - 89)	49 (23 - 103)	0.620
Albumin, g/dl	3.5 ± 0.5	3.8 ± 0.5	3.6 ± 0.5 *	3.4 ± 0.5 *	3.3 ± 0.4 *†	<0.001
CRP, mg/dl	0.0 (0.0 – 1.0)	0.0 (0.0 – 1.0)	0.0 (0.0 – 1.0)	0.0 (0.0 – 1.0)	0.2 (0.0 – 1.0) **	0.022
Creatinine, mg/dl	0.8 ± 0.3	0.7 ± 0.3	0.7 ± 0.2	0.8 ± 0.3	0.8 ± 0.3	0.402
Total cholesterol, mg/dl	190 ± 39	195 ± 44	196 ± 35	183 ± 37 **	190 ± 39	0.032
Hemoglobin, g/dl	11.4 ± 1.5	11.9 ± 1.5	11.4 ± 1.4	11.2 ± 1.4 *	11.2 ± 1.5 *	<0.001

*p<0.01 vs. the non-frail/energy-sufficient group, **p<0.05 vs. the non-frail/energy-sufficient group
†p<0.01 vs. non-frail/energy-shortage group, ‡p<0.05 vs. the non-frail and energy-deficient group
§p<0.01 vs. the frail/energy-sufficient group, ¶p<0.05 vs. frail/energy-sufficient group
BNP: Brain (B-type) Natriuretic Peptide, CFS; Clinical Frailty Scale, CRP; C-reactive protein, FIM: Functional Independence Measure, FOIS: Functional Oral Intake Scale, GNRI: Geriatric Nutritional Risk Index, MNA-SF; Mini Nutritional Assessment-Short Form.

Supplemental Table S3. Rehabilitation outcomes for each group by sex based on Clinical Frailty Scale scores and energy intake classification at discharge.

Men	Total	Non-frail/energy-sufficient group	Non-frail/energy-deficient group	Frail/energy-sufficient group	Frail/energy-deficient group	p
n	202	62	20	77	43	
Energy intake ratio, (%)	95.4 ± 13.4	99.8 ± 2.7	86.5 ± 20.9 *	97.3 ± 12.9 *†	89.6 ± 16.6 *†§	<0.001
Weight, kg	54.5 ± 10.8	56.8 ± 11.3	59.2 ± 10.4	56.4 ± 10.0 †	48.8 ± 9.4 ‡¶	<0.001
Body Mass Index kg/m²	20.8 ± 3.3	21.3 ± 3.3	21.9 ± 2.8	21.0 ± 3.2	19.3 ± 3.1 *	0.004
Handgrip strength, kg	22.5 ± 8.9	27.0 ± 9.7	22.6 ± 8.8	21.4 ± 7.1	18.8 ± 8.0 *‡§	<0.001
Quadriceps strength, kg	17.0 ± 8.1	19.2 ± 8.2	16.0 ± 10.0	16.8 ± 7.4	14.6 ± 8.0	0.066
Thigh circumference, cm	36.7± 5.0	37.9 ± 4.6	39.3 ± 4.5	36.6 ± 4.9	34.2 ± 5.1 *†	<0.001
Calf circumference, cm	30.1 ± 3.9	31.3 ± 3.8	32.0 ± 3.6	29.9 ± 3.5*	27.9 ± 3.7 *†¶	<0.001
FOIS	6.4 ± 1.2	6.9 ± 0.4	6.6 ± 0.8	6.3 ± 1.1 **	5.6 ± 1.8 *‡¶	<0.001
MNA-SF	9.8 ± 2.5	10.7 ± 1.7	10.6 ± 1.7	10.1 ± 2.2	7.8 ± 3.0 *§	<0.001
GNRI	91.1 ± 11.4	95.6 ± 8.3	91.2 ± 10.6	90.0 ± 10.4*	82.8 ± 10.5*	0.017
Energy intake, kcal	1671 ± 368	1871 ± 302	1519 ± 436*	1672 ± 290 *	1452 ± 399 *§	<0.001
Barthel Index, score	90 (75 - 100)	100 (95 - 100)	90 (85 - 100)	90 (70 - 95) *	75 (50 - 90) *†§	<0.001
Changes during hospitalization						
Change in body weight	-0.7 ± 2.7	-0.2 ± 2.8	-0.7 ± 1.9	-0.8 ± 2.7	-1.3 ± 2.8	0.278
Change in handgrip strength	0.6 ± 4.0	0.3 ± 3.4	0.1 ± 3.8	0.9 ± 4.9	0.6 ± 2.7	0.825
Change in quadriceps strength	2.2 ± 9.2	2.5 ± 6.1	0.3 ± 4.3	2.0 ± 6.6	2.8 ± 6.2	0.644
Change in FOIS	0.0 ± 0.8	0.1 ± 0.4	0.0 ± 0.7	0.2 ± 0.7	-0.5 ± 1.7 *§	0.002
Change in MNA-SF	3.2 ± 2.6	3.0 ± 2.6	4.1 ± 2.0	3.3 ± 2.8	3.0 ± 2.5	0.360
Change in GNRI	0.5 ± 3.4	0.8 ± 3.8	0.1 ± 2.3	0.3 ± 0.8	0.4 ± 1.8	0.158
FIM, score						
Motor	77 (63 - 86)	87 (79 - 89)	79 (70 - 85) **	72 (59 - 80) *	66 (35 - 77) *†§	<0.001
Cognitive	28 (21 - 33)	33 (28 - 35)	29 (26 - 34)	25 (19 - 30) *	22 (17 - 28) *†	<0.001
Total	104 (88 - 117)	118 (109 - 123)	107 (93 - 118) **	95 (78 - 108) *	89 (52 - 105) *†§	<0.001
Barthel Index gain, score	30 (15 - 45)	25 (15 - 40)	30 (25 - 45)	35 (20 -45)	35 (15 - 50)	0.393
FIM gain, score						
Motor	34 (25 - 41)	34 (27 - 43)	39 (34 - 42)	34 (26 - 39)	33 (7 - 44) ‡	0.049
Cognitive	3 (0 - 6)	2 (0 - 6)	5 (3 - 6)	3 (0 - 6)	3 (1 - 6)	0.295
Total	37 (27 - 46)	36 (28 - 48)	44 (37 - 49)	36 (27 - 44)	35 (11 -48)	0.062
Length of hospital stay, day	78 (51 - 87)	60 (37 - 79)	75 (52 - 88)	84 (57 - 88) *	83 (61 - 88) **	0.002
FIM efficiency, score/day	0.53 (0.39 – 0.75)	0.63 (0.44 – 0.82)	0.61 (0.50 – 0.87)	0.49 (0.36 – 0.68) **	0.45 (0.14 – 0.68) *	0.002

Women	Total	Non-frail/energy-sufficient group	Non-frail/energy-deficient group	Frail/energy-sufficient group	Frail/energy-deficient group	p
n	533	122	66	153	192	
Energy intake ratio, (%)	92.1 ± 13.0	98.9 ± 4.6	85.8 ± 12.8 *	95.9 ± 9.9 *	86.8 ± 15.6 *§	<0.001
Weight, kg	46.4 ± 8.8	50.2 ± 8.1	47.0 ± 7.6	45.7 ± 9.2 *	44.4 ± 8.4 *	<0.001
Body Mass Index kg/m²	21.2 ± 3.5	22.5 ± 3.5	21.4 ± 3.3 *	20.9 ± 3.6 *	20.6 ± 3.3 *	<0.001
Handgrip strength, kg	13.4 ± 5.5	15.5 ± 5.0	13.5 ± 6.3 *	13.2 ± 6.1 *	12.4 ± 4.4 *	<0.001
Quadriceps strength, kg	13.4 ± 6.3	15.0 ± 6.5	13.8 ± 6.0	13.3 ± 6.5	12.2 ± 6.0 *	0.005
Thigh circumference, cm	35.9± 4.7	37.7 ± 4.4	36.5 ± 4.8	35.4 ± 5.1 *	34.8 ± 4.3 *	<0.001
Calf circumference, cm	29.0 ± 3.5	30.6 ± 3.2	29.1 ± 3.6 **	28.7 ± 3.8 *	28.1 ± 3.1 *	<0.001
FOIS	6.4 ± 1.1	6.8 ± 0.6	6.5 ± 1.1	6.3 ± 1.2 *	6.1 ± 1.2 *	<0.001
MNA-SF	9.6 ± 2.8	11.2 ± 2.2	9.9 ± 2.3 *	9.1 ± 2.9 *	8.9 ± 2.6 **	<0.001
GNRI	91.2 ± 10.7	96.7 ± 10.1	93.9 ± 11.1	87.9 ± 10.6 *‡	88.0 ± 9.0 *‡	<0.001

Supplemental Table S3. Cont.

Women	Total	Non-frail/energy-sufficient group	Non-frail/energy-deficient group	Frail/energy-sufficient group	Frail/energy-deficient group	p
Energy intake, kcal	1447 ± 259	1542 ± 185	1406 ± 268 *	1496 ± 229	1362 ± 288 *§	<0.001
Barthel Index, score	90 (70 - 100)	95 (90 - 100)	90 (85 - 100)	90 (70 - 100) *	80 (60 - 90) *†¶	<0.001
Changes during hospitalization						
Change in body weight	-0.2 ± 2.3	0.1 ± 1.7	-0.6 ± 2.2	-0.2 ± 2.9	-0.3 ± 2.2	0.324
Change in handgrip strength	0.6 ± 3.8	0.8 ± 3.1	-0.2 ± 5.3	0.8 ± 3.2	0.6 ± 4.2	0.347
Change in quadriceps strength	2.4 ± 4.1	2.8 ± 4.5	1.7 ± 4.6	2.7 ± 4.1	1.9 ± 3.6	0.216
Change in FOIS	0.0 ± 0.8	0.0 ± 0.3	0.0 ± 0.9	0.0 ± 0.7	-0.1 ± 1.0	0.832
Change in MNA-SF	2.9 ± 2.6	3.4 ± 2.1	3.2 ± 2.6	2.3 ± 2.4 *	3.0 ± 2.9	0.004
Change in GNRI	0.4 ± 5.4	-0.2 ± 5.7	0.7 ± 5.4	1.0 ± 5.2	0.3 ± 5.3	0.610
FIM, score						
Motor	75 (62 - 83)	83 (74 - 88)	81 (67 -86)	75 (62 -83) *	68 (55 - 77) *†§	<0.001
Cognitive	28 (22 - 33)	33 (28 - 35)	31 (23 - 35)	27 (19 - 33) *	25 (20 - 29) *†	<0.001
Total	102 (83 - 116)	115 (103 - 122)	109 (91 - 120)	100 (82 - 116) *	93 (77 - 104) *†¶	<0.001
Barthel Index gain, score	30 (20 - 45)	25 (15 - 40)	30 (20 - 45)	30 (20 -45)	35 (20 - 50)	0.199
FIM gain, score						
Motor	35(24 - 43)	35 (24 - 41)	36 (29 - 45)	36 (24 - 44)	35 (22 - 43)	0.485
Cognitive	3 (0 - 6)	4 (1 - 6)	3 (0 - 7)	3 (0 - 6)	3 (0 - 6)	0.917
Total	39 (27 - 48)	38 (28 - 46)	41 (31 - 49)	38 (26 - 47)	40 (23 -49)	0.571
Length of hospital stay, day	78 (57 - 87)	63 (44 - 83)	74 (52 - 87)	77 (59 - 87) *	84 (67 - 88) *‡	<0.001
FIM efficiency, score/day	0.54 (0.37 – 0.73)	0.59 (0.44 – 0.83)	0.60 (0.38 – 0.93)	0.54 (0.36 – 0.73) **	0.52 (0.33 – 0.63) *†	<0.001

*p<0.01 vs. the non-frail/energy-sufficient group, **p<0.05 vs. the non-frail/energy-sufficient group
†p<0.01 vs. the non-frail/energy-deficient group, ‡p<0.05 vs. the non-frail/energy-deficient group
§p<0.01 vs. the frail/energy-sufficient group, ¶p<0.05 vs. frail/energy-sufficient group
FIM: Functional Independence Measure, FOIS: Functional Oral Intake Scale, MNA-SF; Mini Nutritional Assessment-Short Form.

Supplemental Table S4. Univariate liner regression analysis and multiple liner regression analysis of rehabilitation effectiveness by sex

Men	Univariate liner regression analysis					Multiple liner regression analysis				
	B	β	p	95%CI		B	β	p	95%CI	
				Lower	Higher				Lower	Higher
						32.711		0.098	-6.074	71.496
Age	-0.751	-0.342	<0.001	-1.038	-0.463	-0.250	-0.118	0.077	-0.527	0.027
CFS	-9.921	-0.409	<0.001	-13.009	-6.833	-5.184	-0.220	0.001	-8.198	-2.171
Energy intake ratio	0.486	0.372	<0.001	0.316	0.655	0.202	0.157	0.020	0.031	0.372
Handgrip strength	1.443	0.518	<0.001	1.095	1.790	0.655	0.233	0.002	0.250	1.060
FOIS	10.268	0.375	<0.001	6.630	13.907	4.856	0.181	0.008	1.260	8.451
MNA-SF	4.573	0.444	<0.00	3.281	5.866	0.948	0.094	0.189	-0.471	2.366
CRP	-3.768	-0.219	0.002	-6.120	-1.415	-2.030	-0.125	0.035	-3.920	-0.140

CFS; Clinical Frailty Scale, CRP; C-reactive protein, FOIS: Functional Oral Intake Scale, MNA-SF; Mini Nutritional Assessment-Short Form.

Women	Univariate liner regression analysis					Multiple liner regression analysis				
	B	β	p	95%CI		B	β	p	95%CI	
				Lower	Higher				Lower	Higher
						5.395		0.773	-31.421	42.211
Age	-1.158	-0.356	<0.001	-1.417	-0.899	-0.391	-0.122	0.004	-0.655	-0.127
CFS	-7.374	-0.325	<0.001	-9.201	-5.546	-2.245	-0.098	0.018	-4.101	-0.388
Energy intake ratio	0.467	0.374	<0.001	0.369	0.566	0.288	0.225	<0.001	0.186	0.389
Handgrip strength	1.995	0.425	<0.001	1.616	2.375	0.992	0.213	<0.001	0.596	1.389
FOIS	11.161	0.413	<0.001	8.974	13.348	4.703	0.168	<0.001	2.353	7.054
MNA-SF	4.414	0.416	<0.001	3.586	5.242	1.571	0.146	0.001	0.660	2.481
BNP	-0.030	-0.111	0.012	-0.053	-0.007	-0.004	-0.015	0.707	-0.023	0.016
Total cholesterol	0.074	0.107	0.013	0.015	0.132	-0.004	-0.005	0.896	-0.058	0.051
Hemoglobin	4.739	0.259	<0.001	3.232	6.247	1.614	0.087	0.036	0.108	3.120

BNP: Brain (B-type) Natriuretic Peptide, CFS; Clinical Frailty Scale, CRP; C-reactive protein, FOIS: Functional Oral Intake Scale, MNA-SF; Mini Nutritional Assessment-Short Form.
